# The Relationship Between the Duration of Attention to Pandemic News and Depression During the Outbreak of Coronavirus Disease 2019: The Roles of Risk Perception and Future Time Perspective

**DOI:** 10.3389/fpsyg.2021.564284

**Published:** 2021-02-11

**Authors:** Lanting Wu, Xiaobao Li, Hochao Lyu

**Affiliations:** ^1^Faculty of Psychology, Southwest University, Chongqing, China; ^2^Time Psychology Research Center, Southwest University, Chongqing, China; ^3^China Community Psychology Service and Research Center, Southwest University, Chongqing, China

**Keywords:** COVID-19, duration of attention to pandemic news, depression, risk perpection, future time perspective

## Abstract

Since the outbreak of coronavirus disease 2019 (COVID-19) in China, people have been exposed to a flood of media news related to the pandemic every day. Studies have shown that media news about public crisis events have a significant impact on individuals' depression. However, how and when the duration of attention to pandemic news predicts depression still remains an open question. This study established a moderated mediating model to investigate the relationship between the duration of attention to pandemic news and depression, the mediating effect of risk perception, and the moderating effect of future time perspective on the relationship. In early February 2020, 701 individuals from 29 provinces, autonomous regions, and municipalities across China were asked to self-report their duration of attention to pandemic news, level of depression, risk perception, and future time perspective during the COVID-19 outbreak. Results show that there is a significant positive correlation between the duration of attention to news on COVID-19 and depression; risk perception mediates the association between the duration of attention to pandemic news and depression; and future time perspective plays a moderating role between risk perception and depression. The findings of the present study provide theoretical implications and practically throw some light on alleviating the public's depression during pandemic periods. We highlight that the individual's hope for a better future, focusing on positive news, and time perspective balance during an epidemic disease are also beneficial to promoting positive emotion and reducing depression.

## Introduction

The pandemic of novel coronavirus disease (COVID-19) is raging across the globe and has become a worldwide public health crisis (Bao et al., 2020). Being confronted with major catastrophic events, the public not only suffers from threats to their lives and safety but also faces psychological impact and even psychological trauma. The occurrence of major disasters has a great adverse impact on people's mental health, leading to negative emotions such as tension, panic, and depression (Hobfoll et al., 2006; Lau et al., 2010). For example, a recent study by Wang et al. (2020), which included 1,210 participants from 194 cities in China during the initial stage of the COVID-19 outbreak, showed that 53.8% of the participants rated the psychological impact of the pandemic as moderate or severe. According to the study of Serafini et al. (2020), various psychological problems and serious consequences in terms of mental health including stress, anxiety, depression, frustration, and uncertainty emerged progressively during this pandemic. More importantly, the public displayed more vicarious traumatization than front-line medical staff (Li et al., 2020). Previous studies have shown that most public perceptions of public crisis events come from the media, which in turn affects public response to those events (Lau et al., 2010). Therefore, the effect of media news on the public's physical and mental health is an issue of particular concern.

As the pandemic was emerging, various news related to the pandemic also exploded. The WHO declared a COVID-19 “infodemic”—“an overabundance of information, some accurate and some not that makes it hard for people to find trustworthy sources and reliable guidance when they need it” (World Health Organization, 2020). Furthermore, cues that are vague, inadequate, unfamiliar, contradictory, numerous, or lacking information prompt uncertainty (Budner, 1962; Mishel, 1984). Therefore, when individuals are faced with a large amount of news about the pandemic, it is difficult for them to distinguish right from wrong and make a judgment. Accordingly, they may feel a sense of uncertainty. Uncertainty is a cognitive state that occurs when one cannot construct events well because certain diseases have many unknown factors (Mishel and Braden, 1988). Uncertain viral transmission may take part a crucial and underestimated role in sustaining the epidemic (Sarkar et al., 2020). Many studies directly linked uncertainty to depression (Mullins et al., 2000; Kang, 2006). Swallow and Kuiper (1992) showed that uncertainty is often associated with depression. When uncertainty was evaluated as a danger, it was generally associated with a pessimistic view of events and the future and resulted in harmful outcomes such as anxiety, depression, and distress (Mishel, 1988). People living in a volatile and insecure environment (e.g., an insecure job, unhappy relationship, poverty, etc.) have a high risk of depression (Mcewen, 1998; Peters and Mcewen, 2015). Therefore, the “infodemic” of pandemic news may bring uncertainty, thus leading to depression. We proposed that, during the pandemic, the more time individuals paid attention to news about the pandemic, the higher the level of depression they might feel. Hence, we predicted that:

H1: The duration of attention to pandemic news will be positively correlated with individuals' depression.

Media news also affect individuals' risk perception (Cooper and Nisbet, 2016; Paek et al., 2016). Research has shown that TV news on a series of public health issues (such as cancer, AIDS, heart disease, and smoking) are positively correlated with risk perception (Paek et al., 2016). The theory of social amplification of risk holds that the interaction of risk information through multiple propagation mechanisms and repeated feedback during the transmission process leads to signal amplification, which then enhances individuals' risk perception (Kasperson and Kasperson, 1996). The agenda-setting theory holds that the media can direct people's attention through the degree of emphasis placed on relevant issues, thereby conveying the key points in the communication of the public agenda. For example, if the media repeatedly communicates risk information related to drug usage, individuals might perceive that the issue has become more and more important. Furthermore, their perceptions of risk, sensitivity, and severity are directly related to the degree of media coverage of drug usage (Gelders et al., 2009). In summary, it could be inferred that during the pandemic of COVID-19, various real-time pandemic news reports and related information have been communicated frequently and repeatedly to the public through multiple transmission mechanisms, which caused the public's risk perception to rise dramatically. Accordingly, this study predicted:

H2: The duration of attention to pandemic news will be positively related to risk perception.

In addition, risk perception, as an important variable affecting mental health, refers to a subjective assessment of the likelihood of threat events (Slovic, 1987; Li et al., 2009). Brewer et al. (2007) proposed that risk perception includes three components: uncertainty (probability of being injured by danger), susceptibility (physical vulnerability in the face of danger), and severity (degree of harm caused by danger). Studies on the SARS epidemic and Wenchuan earthquake in China have shown that risk perception is not conducive to people's mental health (Shi et al., 2003; Li et al., 2009). COVID-19 has become a stressor for many people (Li et al., 2020). Lazarus and Folkman (1984) proposed that the consequences of environmental stressors on health depended on the assessment of threats (primary assessment) and the assessment of available personal resources for responding to threats (secondary assessment). Thus, individuals will have a risk perception of the pandemic that is full of uncertainties and a risk perception of uncontrollable and involuntary exposure; such unavoidable situations with loss of control for negative events cause individuals to develop depression disorders (Abramson et al., 1978). As susceptibility (a kind of diathesis) is a negative cognition, the hopelessness theory of depression (Abramson et al., 1989) holds that when exposed to negative life events, people who show negative inferential styles are more likely to make negative judgments when exposed to stressful events and thereby have an increased likelihood of experiencing depression. The severity of public crisis events can also aggravate an individual's depression. For example, during the SARS outbreak, the level of depression and anxiety among medical staff was higher than that during the prevention period (Chen et al., 2006).

In conclusion, the duration of attention to pandemic news would motivate individuals' depression and risk perception, which, in turn, would also lead to depression. With this view in mind, this study proposed the following predictions:

H3: Risk perception will be positively correlated with individuals' depression.
H4: Risk perception plays a mediating role in the relationship between the duration of attention to pandemic news and depression.

Social cognitive theory holds that, as an important factor affecting mental health (Bandura, 1997; Henshaw and Freedman-Doan, 2009), individual belief is a psychological resource that effectively responds to environmental threats (Keller et al., 2011). Future time perspective (FTP), as a belief for the future, is a relatively stable personality trait that represents individual's cognition of, emotional experience of, and action (action tendency) toward the future (Huang, 2004; Lyu, 2014). Individuals with higher FTP are more optimistic about the future, more confident in achieving future goals, and firmly believe that their current behaviors will help in achieving future goals (Kooij et al., 2018). FTP helps individuals achieve self-adjustment by postponing gratification, and individuals with high FTP could perceive the higher value of delayed rewards so that they are able to focus on the future even in the absence of immediate rewards (Bembenutty and Karabenick, 2004; Lyu and Huang, 2016).

FTP has also been shown to be related to mental disorders (Kooij et al., 2018). In the face of adversity, individuals with higher FTP have greater adaptability and show less depression (Epel et al., 1999), and adolescents with longer FTP have lower depression levels (Diaconu-Gherasim et al., 2017). Conversely, individuals with lower FTP are less clear and more pessimistic about the future and may have greater worries and anxieties about an unpredictable and uncertain future. With the individual's perception of time being as one of the potential predictors of PTSD, traumatic events may cause the individuals to lose expectations for the future, to sink into their current pain and past experience, which can then place their mental health at risk (Holman et al., 2016). The core concept of cognitive behavioral therapy of depression—the “cognitive triad” model of depression—also includes individuals' negative views of the future. The theory holds that for individuals who have negative attitude toward their own abilities and believe that success is impossible and life has no hope, such negative views of the future will lead to depression (Beck and Weishaar, 1989). This suggests that FTP may be an important factor affecting individuals' depression during the pandemic. Allemand et al. (2012) demonstrated the moderating role of FTP in the relationship between tolerance and subjective well-being, and those who believed that the future was open-ended tended to report more life satisfaction and less pessimism than those who perceived their future as restricted. Therefore, FTP may play a moderating role in the relationship between individuals' risk perception and depression. Studies by Andre et al. (2018) and De Volder and Lens (1982) show that those who believe the future is open-ended tend to score higher in positive emotions and in the sense of life and lower in negative emotions (Hicks et al., 2012). Therefore, higher FTP may help facilitate positive emotions of individuals, ease negative emotions under a stressful environment of risk perception, and then mitigate depression. Hence, this study predicted:

H5: FTP may play a moderating role in the relationship between risk perception and depression.

**Figure 1:**
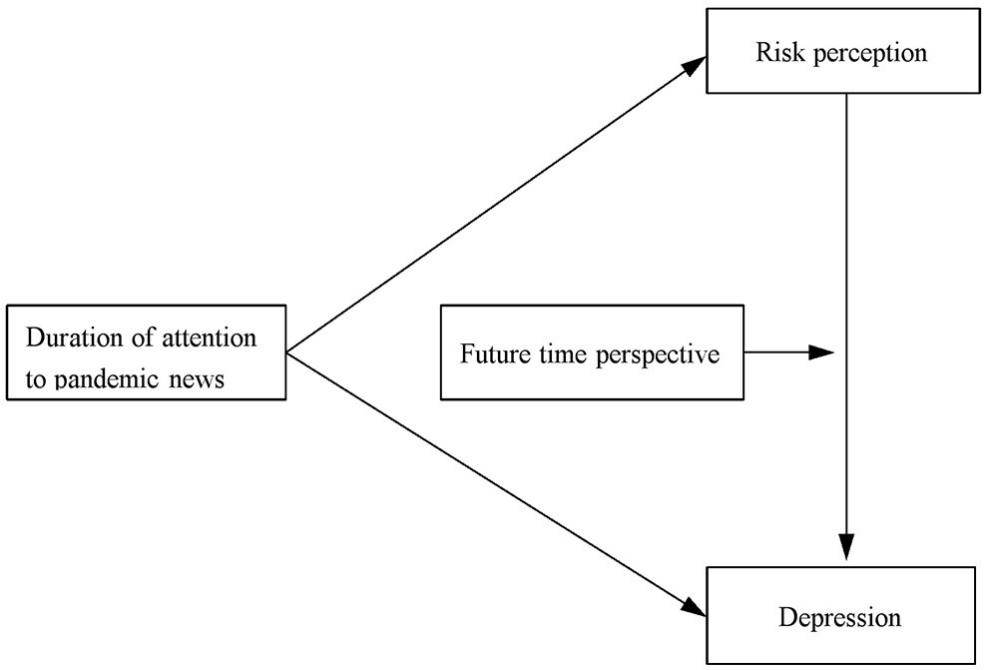
The proposed moderated mediation model.

In sum, the current study aims to investigate the mediating effect of risk perception in the relationship between the duration of attention to pandemic news and depression during the outbreak of COVID-19, as well as the moderating effect of FTP on this mediating effect. The hypothetical model is shown in Figure 1.

## Materials and Methods

### Participants and Procedure

Due to the infectivity of the novel coronavirus, this study adopted a cross-sectional online survey (in Mandarin) with Survey Star (wjx.cn), a platform providing functions equivalent to Amazon Mechanical Turk. The questionnaires were released on February 6th, 2020, through WeChat platform during the outbreak of COVID-19 in China. As of February 15, 2020, 701 participants from China completed the questionnaires. All participants consented to participate in the study after being informed about the purpose of the study, and the investigation was approved by the Faculty of Psychology at Southwest University. Among all participants, 37.8% (*n* = 265) were males and 62.2% (*n* = 436) were females. As for age, 1.4% were under the age of 18, 28.8% were between 18 and 25 years old, 18.0% were between 26 and 30 years old, 22.5% were between 31 and 40 years old, 21.1% were between 41 and 50 years old, 7.6% were between 51 and 60 years old, and 0.6% were over 60 years old.

### Materials

#### Duration of Attention to Pandemic News

One single item “How long do you browse, read or watch the pandemic news every day?” was used to collect the participants' daily attention to the pandemic news. Ratings were given on an eight-point scale with higher scores indicating longer duration of attention to pandemic news. The distribution of participants' duration of attention to pandemic news is shown in Table 1.

**Table 1:** Distribution of participants' duration of attention to pandemic news.

**Rate**	***N* (%)**
1 = no attention	4 (0.6)
2 = within 10 min	58 (8.3)
3 = 10 to 30 min	167 (23.8)
4 = 0.5 to 1 h	145 (20.7)
5 = 1 to 2 h	180 (25.7)
6 = 3 to 5 h	120 (17.1)
7 = 6 to 9 h	17 (2.4)
8 = more than 10 h	10 (1.4)

#### Risk Perception of the Pandemic

We developed a six-item scale to measure individuals' risk perception of COVID-19 according to the study by Brewer et al. (2007). Items are as follows: “I am very worried that my family and I will be infected with the novel coronavirus (uncertainty)”; “I feel that washing my hands and wearing a mask cannot stop the invasion of the novel coronavirus (susceptibility)”; “I think the actual numbers of people infected are much higher than the official figures (severity)”; “I am afraid that I will be infected with the novel coronavirus (uncertainty) when I go out”; “When it is necessary to go out (such as purchasing daily necessities), I will be far away from the crowds (serious impact on life)”; and “I am afraid to touch things touched by others, such as supermarket receipts, elevator buttons, etc. (serious impact on life).” All items were rated on a five-point Likert scale (1 = strongly disagree to 5 = strongly agree). Higher scores reflect an increased perceived risk of the pandemic situation. The Cronbach's alpha score of the scale was 0.74.

#### Future Time Perspective

The Chinese version of the ZTPI Future Subscale (Lyu et al., manuscript under review; Zimbardo and Boyd, 1999) was used to measure FTP. This scale contains five items, rated on a five-point Likert scale (from 1 = very uncharacteristic to 5 = very characteristic). Sample items were as follows: “When I want to accomplish something, I will set a goal and consider specific ways to achieve it” and “I can usually complete the plan step by step.” In this study, the Cronbach's alpha score of the scale was 0.86.

#### Depression

We adopted the Center for Epidemiological Studies Depression scale (CES-D; Andresen et al., 1994) to assess depressive symptoms. The CES-D has 10 items, which are rated on a four-point scale from 0 (rarely or none of the time) to 4 (most or all the time) based on how often participants felt that way in the previous 2 weeks, with higher scores indicating more depression. Sample items were as follows: “Even though my family and friends helped me, I couldn't get rid of my depression” and “I feel that my life is a failure.” The Cronbach's alpha in this study was 0.84.

### Statistical Analysis

IBM SPSS Statistics 22.0 was used for data entry, sorting, and analysis. We used model 4 of the Hayes (2017) PROCESS macro to examine the mediation effect of risk perception and model 14 to examine the moderating effect of FTP. Bootstrapping (5,000 bootstrap samples) with 95% confidence intervals (CIs) was conducted to test the significance of indirect effects (Hayes, 2017). The 95% CIs did not include zero, indicating a significant effect.

## Results

### Correlations Analysis

Table 2 shows the means, standard deviations, and correlation matrix of each variable. As expected, the duration of attention to pandemic news was positively correlated with risk perception (*r* = 0.13, *p* < 0.01) and depression (*r* = 0.11, *p* < 0.01). Also, there were a significant positive correlation between risk perception and depression (*r* = 0.26, *p* < 0.01) and a significant negative correlation between FTP and depression (*r* = 0.32, *p* < 0.01). These results provided preliminary support for our hypotheses. Moreover, gender and age were associated with duration of attention to pandemic news, risk perception, depression, and FTP, so they were included as control variables in the following analysis.

**Table 2:** Means, standard deviations, and correlations.

**Variable**	***M***	**SD**	**1**	**2**	**3**	**4**	**5**	**6**
1. Duration of attention to pandemic news	4.33	1.37	1					
2. Risk perception	3.56	0.73	0.13^**^	1				
3. Depression	1.83	0.48	0.11^**^	0.26^**^	1			
4. FTP	3.62	0.71	0.07	0.03	−0.32^**^	1		
5. Gender	1.62	0.49	−0.04	0.10^*^	0.04	−0.05	1	
6. Age	3.58	1.36	0.17^**^	0.03	−0.16^**^	0.25^**^	−0.25^**^	1

*N = 701. Internal consistency reliabilities in the diagonal. Gender: 1 = male, 2 = female. Age: 1 = under 18, 2 = 18 to 25, 3 = 26 to 30, 4 = 31 to 40, 5 = 41 to 50, 6 = 51 to 60, 7 = over 60*.

* *p < 0.05*;

** *p < 0.01 (two tailed)*.

### Mediation Analysis

We used a series of regression analysis to test our hypotheses. As shown in Table 3, after the effects of gender and age had been controlled, the duration of attention to pandemic news emerged as positively related to both risk perception (*B* = 0.13, SE = 0.04, *p* < 0.01, model 1) and depression (*B* = 0.14, SE = 0.04, *p* < 0.01, model 3), thereby supporting Hypotheses 1 and 2. When controlling for the duration of attention to pandemic news, risk perception was positively related to depression (*B* = 0.26, SE = 0.04, *p* < 0.01, model 2), so Hypothesis 3 was supported. Moreover, bootstrapping indicated that the mediation effect of risk perception was significant [*B* = 0.03, SE = 0.01, Boot 95% CI (0.01, 0.06)]. Taken together, risk perception mediated the relationship between the duration of attention to pandemic news and depression. Thus, Hypothesis 4 was supported.

**Table 3:** Testing the mediation effect of risk perception on depression.

**Predictor variable**	**Model 1 (risk perception)**	**Model 2 (depression)**	**Model 3 (depression)**
	***B* (SE)**	***B* (SE)**	***B* (SE)**
Gender	0.12 (0.04)^**^	−0.03 (0.04)	−0.02 (0.04)
Age	0.04 (0.04)	−0.19 (0.04)^***^	−0.18 (0.04)^***^
Duration of attention to pandemic news	0.13 (0.04)^***^	0.10 (0.04)^**^	0.14 (0.04)^***^
Risk perception		0.26 (0.04)^***^	
*R*^2^	0.03	0.11	0.04
*F*	6.94	20.72	9.88

*N = 701. Bootstrap sample size = 5,000*.

*B, unstandardized regression coefficients*.

*p < 0.05;

** *p < 0.01*;

*** *p < 0.001*.

### Moderated Mediation Analysis

**Figure 2:**
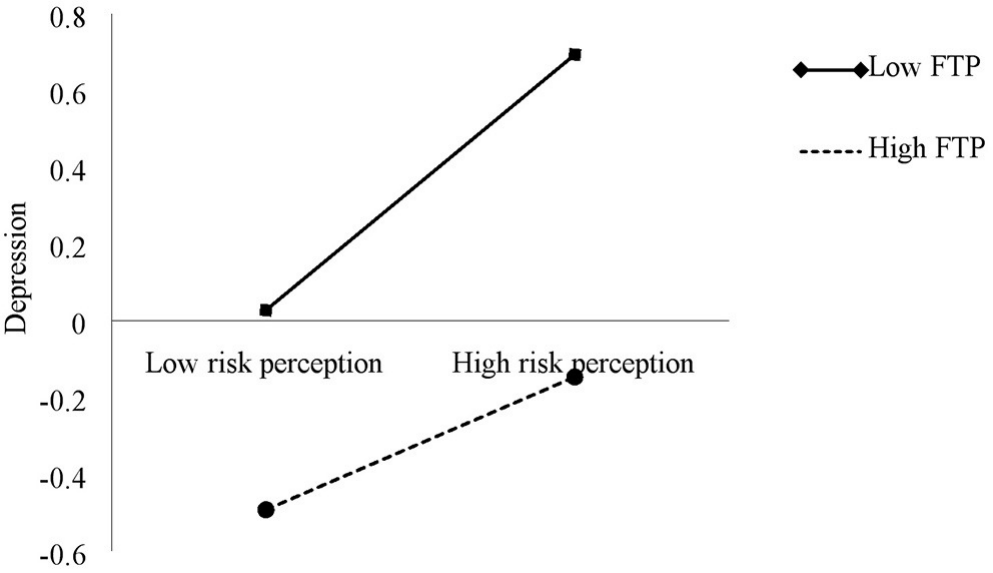
The interactive effect of future time perspective and risk perception on depression.

Controlling for age and gender, we conducted the moderated mediation analysis. As shown in Table 4, the interaction (model 5) between risk perception and FTP significantly predicted depression (*B* = −0.07, SE = 0.03, *p* < 0.05), suggesting that FTP moderated the effect of risk perception on depression. In order to more clearly interpret the interactive effect of FTP and risk perception on depression, FTP scores were divided into high and low groups according to plus or minus one standard deviation. We conducted a simple slope analysis to examine the predictive effect of risk perception on depression at high and low FTP levels. Simple slope tests (Figure 2) suggested that for individuals with high FTP level, risk perception has a significant predictive effect on depression [*B* = 0.20, SE = 0.05, 95% CI (0.10, 0.29)]; for individuals with low FTP level, risk perception is also a significant predictor of depression [*B* = 0.33, SE = 0.05, 95% CI (0.24, 0.41)]. The positive effects of risk perception on depression were stronger for those respondents with low FTP. It was found that the interaction term between FTP and risk perception significantly predicted depression. For depression, the index of moderated mediation was −0.01 [95% CI (−0.02, −0.00), excluding zero]. When the level of FTP was high, the duration of attention to pandemic news had a significant indirect effect on depression through risk perception [*B* = 0.03, SE = 0.01, 95% CI (0.01, 0.05)]; when the level of FTP was low, the duration of attention to pandemic news also had a significant indirect effect on depression through risk perception [*B* = 0.04, SE = 0.02, 95% CI (0.01, 0.07)]. The positive effects of the duration of attention to pandemic news on depression through risk perception were stronger for those respondents with low FTP. Thus, Hypothesis 5 was supported.

**Table 4:** Testing the moderated mediation effect of FTP on depression.

**Predictor variable**	**Model 4 (risk perception)**	**Model 5 (depression)**
	***B* (SE)**	***B* (SE)**
Gender	0.12 (0.04)^**^	−0.03 (0.04)
Age	0.04 (0.04)	−0.11 (0.04)^**^
Duration of attention to pandemic news	0.13 (0.04)^***^	0.11 (0.04)^**^
Risk perception		0.26 (0.04)^***^
FTP		−0.32 (0.04)^***^
Risk perception ^*^ FTP		−0.07 (0.03)^*^
*R*^2^	0.03	0.20
*F*	6.94	29.31

*N = 701. Bootstrap sample size = 5,000*.

*B, unstandardized regression coefficients*.

* *p < 0.05*;

** *p < 0.01*;

*** *p < 0.001*.

## Discussion

In this study, we found that there was a significant positive correlation between the duration of attention to pandemic news and depression. Xu and Sattar (2020) also found that there was a positive correlation between pandemic information from media and panic, which indicates that the news of the pandemic has an important impact on individuals' physical and mental health. During the pandemic, the public generated a lot of negative emotions, such as panic and depression. Specifically, since COVID-19 is an emerging, infectious, and unknown disease, there is a lack of knowledge about it. Under the implementation of the isolation measures, the public mostly formed their perception and understanding of the pandemic through news media. Faced with the “infodemic” of pandemic news, on the one hand, it is difficult for individuals to judge the authenticity of the news and form an accurate cognition. On the other hand, in the early stages of the pandemic, many contradictory news in the media led people to doubt the valid information. They also even doubted the official data because of the prevailing rumors (Xu and Sattar, 2020). Therefore, surrounded by various pandemic news, it is difficult for people to find trustworthy sources and reliable guidance. With individuals getting more news about the pandemic, they still cannot accurately understand the epidemic itself, its consequences, and its prevention and control, which lead them to feel uncertainty (Xu and Sattar, 2020). Uncertainty, as we all know, is one of the important factors that lead to depression (Swallow and Kuiper, 1992). Therefore, as H1 predicted, the duration of attention to pandemic news is positively correlated with individuals' depression.

Few studies have been done to investigate the psychological mechanism of the effect of duration of attention to pandemic news on depression. The current study showed that the duration of attention to pandemic news has a predictive effect on people's risk perception, and according to the theory of social amplification of risk and agenda setting, the duration of attention to pandemic news will increase people's risk perception (Gelders et al., 2009; Paek et al., 2016). For example, when receiving reports of the rapid growth of confirmed cases from the media, which exacerbates individuals' risk perception, people may overestimate their possibility of infection on the one hand and put in place more protective measures on the other hand, such as wearing masks, washing hands frequently, and staying away from crowds, which are all behaviors that follow after intensified risk perception. Behind these behaviors, the media's coverage of tragic events has resulted in people's emotional changes, which has caused them to frequently overestimate the frequency of risks and other adverse events (Johnson and Tversky, 1983). From the perspective of the dual process theories, emotion is exactly the basis of a heuristic system, and relying on intuitive emotions is a faster, easier, and more effective way of judgment, especially in complex, uncertain, and even dangerous situations (Slovic and Peters, 2006). Therefore, individuals often use initial emotional impressions of information to judge risk (Cooper and Nisbet, 2016). By this line of reasoning, the emotions conveyed *via* media reports are important factors influencing individuals' risk perception. News information that triggers fear is more likely to intensify individuals' level of risk perception. Thus, there is a positive correlation between fear and risk perception (Paek et al., 2016). Moreover, when the media reports contain emotional vocabulary, they may create an alternative experience, causing similar emotional experiences between the public and the victim (Kupchik and Bracy, 2009). Therefore, the emotional information (such as fear and alternative experiences) conveyed by news media during the pandemic will affect public's perception of risks.

The present study showed that risk perception also had a significant direct predictive effect on depression, which is consistent with the hopelessness theory (Abramson et al., 1989) and the diathesis–stress model (Monroe and Simons, 1991). First of all, the possibility of danger is uncertain. When an individual has not developed a cognitive schema for the disease event, vague, insufficient, and unfamiliar information helps the individual perceive an uncertainty and, thus, cannot accurately predict the results. Uncertainty is considered to be a risk, not an opportunity; with risk assessment positively correlated with depression and opportunity assessment negatively correlated with depression, uncertainty affects depression through risk assessment (Kang, 2006). The hopelessness theory (Abramson et al., 1989) points out that when people are exposed to negative life events, those who exhibit negative inferential styles are more likely to suffer from depression. The diathesis–stress model also proposes that stress may activate an individual's diathesis or vulnerability, transforming potential diathesis into a reality of psychopathology, and thus, the impact of stress on depression risk depends on diathesis (Monroe and Simons, 1991). Amid this pandemic, the individuals' perception of the uncertainty of infection, their diathesis, and the serious consequences that may occur after infection have affected their levels of depression. Therefore, risk perception plays a mediating role between the duration of attention to pandemic news and depression. As individuals spend more time being exposed to pandemic news, their risk perception is also higher, which in turn leads to an increase in the levels of depression.

We also found that FTP plays a moderating role in the indirect relationship between the duration of attention to pandemic news and depression, and FTP can buffer the impact of risk perception triggered by the duration of attention to pandemic news on depression. As FTP increases, the indirect effect of the duration of attention to pandemic news on depression through risk perception weakens. The motivational role of FTP is not only reflected on the cognitive and behavioral levels but also on the emotional level. The more optimistic individuals are about the future, the more confident they are in achieving future goals (Shipp et al., 2009). These positive feelings about the future can alleviate the effects of negative emotions caused by risk perception on depression. Moreover, the greater an individual's risk perception, the greater the pressure will be (Webster et al., 1988). Zhao et al. (2020) found that during the COVID-19 pandemic, perceived stress was positively associated with anxiety. The level of FTP can affect an individual's pressure perception (Fooken, 1982). The weaker an individual's feeling of time control, the greater the perceived pressure (Nonis et al., 1998). Therefore, higher levels of FTP can alleviate the pressure caused by an individual's perception of risks and thereby reduce the level of depression. In a word, FTP plays a negative moderating role in the indirect relationship between the duration of attention to pandemic news and depression.

The results of this study provide insight for reducing the level of depression among the public during the pandemic and in other major emergencies in the future. First, the findings show that the information conveyed by news reports could affect an individual's level of depression. This conclusion helps to understand the impact of media exposure on an individual's psychological distress during major crisis events, which provides valuable contributions to the ongoing improvement of news media work. On the one hand, the official information helps to reduce people's concerns about the continued development of the situation. For example, previous research shows that positive news information after an earthquake has a protective effect against the continued development of depression and suicidal thoughts (Lau et al., 2010). Moreover, Khajanchi and Sarkar (2020) apply the model to forecast the development of COVID-19 pandemic in India, which suggests that media effect can play a key role in mitigating the transmission of COVID-19. By this line of reasoning, the media should not only report quickly and accurately but also balance the coverage of positive and negative news. On the other hand, the duration of attention to pandemic news may lead to uncertainty, which aggravates individuals' depression. Zhang et al. (2020) have shown that participants who spent more time watching TV and on cellphones or computers were significantly more likely to report elevated stress; those who participated in family entertainment activities were significantly less likely to report elevated stress. Therefore, when the news surges, individuals should attempt to reduce their attention to it and divert their attention to other topics or activities (such as watching entertainment programs, developing hobbies and interests, upgrading personal skills, and spending quality time with family) aimed at alleviating negative emotions. Second, accurate understanding of risks is also an important way to maintain mental health. Individuals should pay attention to official information from formal channels and should not make unfounded speculations or exaggerate the severity of the pandemic and other events. Individuals should also develop an objective and scientific understanding of the pandemic situation, reduce the perceived uncertainty, and develop a sense of control. In order to mitigate uncertainty, individuals should seek to change negative perceptions and develop positive coping behaviors and, thus, reduce risk perception and tendency toward depression. Finally, FTP shows a negative moderating role in the indirect relationship between the duration of attention to pandemic news and depression. Given the plasticity of FTP (Kooij et al., 2018), it is very important to intervene with FTP. Specifically, individuals should be trained to shift their time focus, engage in more positive future thinking, and enhance the role of positive emotions. With the negative news environment surrounding the pandemic, individuals can be instructed to extend their timeline to the future and feel the malleability of the future in order to balance the current negative emotions. The individual's good hope for the future and the confidence in winning the battle against COVID-19 are conducive to promoting positive emotion and reducing depression caused by the negative emotion under the pandemic.

This study has some limitations that should be considered. First, due to its cross-sectional nature, this study could not determine the causal relationship between variables by longitudinally tracking changes during the pandemic. It is suggested that future research should examine causal relationships among variables through a longitudinal study. Second, the study measured the duration of attention to pandemic news but did not distinguish among negative, positive, and neutral information; different types of information may have different effects on risk perception. For example, among the negative information that is closely related to individuals, information concerning whether there is a confirmed case in the same community can most affect the individuals' risk perception (Shi et al., 2003). Therefore, future research needs to explore the impact of different types of information on risk perception and their internal mechanisms. Third, the sample selection was biased, as the sampling did not take into account a balance of different regions. The severity of the pandemic varied greatly in different regions of the country; thus, we should be cautious about generalizing the research results to other regions. Finally, this study did not examine whether individual FTP is also affected by the pandemic, as the view of time will change with personal and situational factors (Kooij et al., 2018). Therefore, future research should further test the interaction and effect of FTP and pandemics.

## Conclusion

The current study found that there is a significant positive correlation between the duration of attention to pandemic news and depression, and risk perception plays a mediating role in the relationship between them. FTP plays a moderating role between risk perception and depression, and individuals with high FTP have a weaker positive effect on depression than those with low FTP.

## Data Availability Statement

The raw data supporting the conclusions of this article will be made available by the corresponding authors, without undue reservation.

## Ethics Statement

The studies involving human participants were reviewed and approved by Faculty of Psychology at Southwest University. Written informed consent to participate in this study was provided by the participants' legal guardian/next of kin.

## Author Contributions

HL designed the research framework and contributed to manuscript writing and modification. LW contributed to the data analysis and writing. XL contributed to the data collection and manuscript modification. All authors contributed to the article and approved the submitted version.

## Conflict of Interest

The authors declare that the research was conducted in the absence of any commercial or financial relationships that could be construed as a potential conflict of interest.
